# The usefulness of ^18^F-FDG PET/CT for assessing methotrexate-associated lymphoproliferative disorder (MTX-LPD)

**DOI:** 10.1186/s12885-016-2672-8

**Published:** 2016-08-15

**Authors:** Shiro Watanabe, Osamu Manabe, Kenji Hirata, Noriko Oyama-Manabe, Naoya Hattori, Yasuka Kikuchi, Kentaro Kobayashi, Takuya Toyonaga, Nagara Tamaki

**Affiliations:** 1 Department of Nuclear Medicine, Hokkaido University Graduate School of Medicine, N15 W7, Kita-Ku, Sapporo, 0608638 Hokkaido Japan; 2 Department of Diagnostic and Interventional Radiology, Hokkaido University Graduate School of Medicine, N15 W7, Kita-Ku, Sapporo, 0608638 Hokkaido Japan

**Keywords:** Methotrexate-associated lymphoproliferative disorder, FDG, PET, Metabolic tumor volume, Total lesion glycolysis

## Abstract

**Background:**

Methotrexate-associated lymphoproliferative disorder (MTX-LPD) is a benign lymphoid proliferation or malignant lymphoma in patients who have been treated with MTX. MTX withdrawal and observation for a short period should be considered in the initial management of patients who develop LPD while on MTX therapy. Here we evaluated the diagnostic accuracy and predictive value of ^18^F-fluorodeoxyglucose positron emission tomography/computed tomography (^18^F-FDG PET/CT) for MTX-LPD.

**Methods:**

We retrospectively investigated the cases of 15 patients clinically suspected of having MTX-LPD. A total of 324 anatomic regions (207 nodal and 117 extranodal regions) were assessed by ^18^F-FDG PET/CT and by multi-detector row CT (MDCT). Each anatomic region was classified as either malignant or benign. The uptake of ^18^F-FDG was assessed semi-quantitatively with the standardized uptake value maximum (SUVmax), the whole-body metabolic tumor volume (WBMTV), and the whole-body total lesion glycolysis (WBTLG) in order to investigate predictive factors of spontaneous regression after the withdrawal of MTX.

**Results:**

MTX-LPD lesions were observed in 92/324 (28.4 %) regions. ^18^F-FDG PET/CT showed 90.2 % sensitivity, 97.4 % specificity, and 95.4 % accuracy, values which were significantly higher than those of MDCT (59.8, 94.8, and 84.9 %, respectively. *p* < 0.002). After the withdrawal of MTX, 9/15 patients (60.0 %) achieved complete response (CR). The SUVmax, WBMTV and WBTLG values of the CR patients were 9.2 (range 2.8–47.1), 44.3 (range 0–362.6) ml, 181.8 (range 0–2180.9) ml, respectively, which were not significantly different from those of the non-CR patients: 10.6 (range 0–24.9), 15.7 (range 0–250.1) ml, and 97.4 (range 0–1052.1) ml.

**Conclusions:**

Although ^18^F-FDG PET/CT was a useful tool to detect MTX-LPD lesions, none of the ^18^F-FDG PET parameters before the withdrawal of MTX could be used to predict CR after the withdrawal of MTX.

## Background

Methotrexate-associated lymphoproliferative disorder (MTX-LPD) is a rare benign lymphoid proliferation or malignant lymphoma observed in patients who have been treated with MTX. It was first reported in 1991 [1] and is now defined by the World Health Organization (WHO) as among the “immunodeficiency-associated LPDs” [2]. MTX is typically administered as a treatment for inflammatory diseases, especially rheumatoid arthritis (RA), and it has been an anchor drug in the treatment of RA. There are many reports and case series of MTX-LPD spontaneously regressing shortly after the discontinuation of MTX [3–6]. It was also reported that only histology and Epstein-Barr virus (EBV) positivity were useful for predicting clinical outcomes in patients with RA-LPD [7].

^18^F-fluorodeoxyglucose (^18^F-FDG) positron emission tomography/computed tomography (PET/CT) is an indispensable tool that can detect metabolically active disease in the whole body, with increased diagnostic accuracy in malignant disease. Thus, ^18^F-FDG PET/CT has been reported to provide superior information for the staging of non-Hodgkin lymphoma (NHL) compared to conventional CT scans [8–10]. The volume-based parameters from ^18^F-FDG PET, such as the maximum standardized uptake value (SUVmax), the whole-body metabolic tumor volume (WBMTV) and whole-body total lesion glycolysis (WBTLG) have been reported to be useful for making a detailed prediction of the prognosis in patients suffering from various types of lymphoma [11–14]. These studies indicated that high metabolic activity might be useful when considering candidates for aggressive therapy and for identifying patients at increased risk for relapse after therapy. However, there have been very limited reports about a clinical role of ^18^F-FDG PET/CT in MTX-LPD [4], and no study has examined the relationships between the prognosis of MTX-LPD and ^18^F-FDG PET parameters such as SUVmax, WBMTV, and WBTLG.

The treatment for immunodeficiency-associated LPD in patients treated with MTX is to stop the immunosuppressive condition by discontinuing the MTX, which differs from the treatment for NHL. Although no strict guidelines for the treatment of MTX-LPD have been established, the withdrawal of MTX and the use of observation-alone have usually been considered to be beneficial [7, 15–17]. Unfortunately, there is a subgroup of patients who do not respond to MTX withdrawal and need cytotoxic chemotherapies. Being able to predict spontaneous regression before the withdrawal of MTX would be greatly beneficial, as unnecessary cytotoxic therapies could then be avoided.

The first purpose of the present study was to investigate the diagnostic accuracy of ^18^F-FDG PET/CT compared to that of multidetector row CT (MDCT) for MTX-LPD. The second purpose was to determine whether ^18^F-FDG PET parameters could predict a complete response (CR) after the withdrawal of MTX.

## Methods

### Subjects

This retrospective study was approved by the institutional review board (#013-0422).

We retrospectively analyzed the imaging reports of the patients suspected of having MTX-LPD who underwent PET/CT scanning at Hokkaido University Hospital during the period from June 2009 to December 2014. We searched the electronic database of all PET/CT scans performed during this time period to identify the patients suspected of having MTX-LPD, and the clinical or histological diagnosis was confirmed by review of each patient’s electronic medical records.

A total of 26 patients suspected of having MTX-LPD were identified by the review. The diagnosis of MTX-LPD was made in 22 of these patients. Regarding the other three patients, they were diagnosed with other pathologies (i.e., lung cancer, chronic lymphocytic leukemia, reactive lymph node in one patient each), and the association between MTX treatment and the remaining diagnosed plasma cytoma was unclear; these patients were therefore excluded from the analyses. We also excluded the cases of five patients who were followed up or received additional therapy at another hospital and two patients whose MTX was withdrawn before FDG PET/CT. The final study population was 15 patients with MTX-LPD (62.0 ± 10.7 years old, five men, ten women). Their characteristics are summarized in Table 1. The reasons for MTX therapy were RA (*n* = 13), polyarteritis nodosa (*n* = 1), and psoriatic arthritis (*n* = 1). The mean duration of MTX treatment was 84.1 ± 59.6 months (range 7–234 months). Nodal regions of lymphoma were observed in 11 of the 15 patients. Extranodal regions of lymphoma were observed in eight of the patients, including the gingiva (*n* = 2), skin/subcutaneous site (*n* = 4), lung (*n* = 4), liver (*n* = 2), pericardium (*n* = 1), bowel (*n* = 1), bone (*n* = 1), tonsil (*n* = 1) and adrenal (*n* = 1).

**Table 1:** Characteristics of the 15 patients with MTX-LPD

No	Age range	Underlying disease	Length of MTX (mo)	Final dose (mg/wk)	sIL-2R (U/ml)	LDH (U/L)	PS	Histological type	EBV infection	Stage	IPI	Prognosis
1	70s	RA	48	8	259	212	0	FL	-	I	1	non-CR
2	70s	RA	138	6	5861	190	3	DLBCL	-	IV	4	non-CR
3	60s	RA	234	8	3744	292	3	-	+	III	4	non-CR
4	70s	RA	103	6	310	241	1	MALT lymphoma	N/A	II	2	CR
5	60s	PN	40	15	670	254	1	DLBCL	+	IV	4	non-CR
6	50s	PA	83	10	3157	336	2	-	+	IV	3	CR
7	50s	RA	101	10	737	229	0	polymorphic BLPD	-	III	1	non-CR
8	70s	RA	50	8	864	209	1	polymorphic BLPD	+	IV	3	CR
9	70s	RA	13	4	503	221	1	DLBCL	-	IE	1	CR
10	40s	RA	7	8	758	149	0	-	N/A	II	0	CR
11	50s	RA	48	6	340	176	1	polymorphic BLPD	+	IIE	0	CR
12	40s	RA	48	16	1370	192	1	-	-	III	1	CR
13	60s	RA	116	8	1136	242	1	DLBCL	-	IV	3	CR
14	50s	RA	60	16	852	231	1	MZL	+	IV	3	non-CR
15	60s	RA	172	8	402	252	0	DLBCL	+	IV	4	CR

*BLPD* B-cell lymphoproliferative disease, *CR* complete response, *DLBCL* diffuse large B-cell lymphoma, *EBV* Epstein-Barr virus, *F* female, *FL* follicular lymphoma, *IPI* International Prognosis Index, *LDH* lactate dehydrogenase, *M* male, *MTX* methotrexate, *MZL* marginal zone lymphoma, *N*/*A* not available, *PA* psoriatic arthritis, *PN* polyarteritis nodosa, *PS* performance status, *sIL*-*2R* soluble interleukin-2 receptor

Pathological data were obtained using tissue specimens obtained by biopsy or resection from 11 patients. The other four patients were diagnosed based on their clinical course. The pathologically confirmed histological types were diffuse large B-cell lymphoma (*n* = 5), pleomorphic B-cell lymphoproliferative disease (*n* = 3), follicular lymphoma (*n* = 1), mucosa-associated lymphoid tissue lymphoma (*n* = 1), and marginal zone lymphoma (*n* = 1). After the withdrawal of MTX, nine of the 15 patients (60.0 %) achieved a CR, and the other six patients (40.0 %) were non-CR. The mean follow-up duration was 31.7 ± 19.4 months (range 9–67 months).

### Assessment of clinical information

The histopathological diagnosis was determined on the basis of the WHO lymphoma classification criteria [2] for the 11 patients whose pathological data were available. In addition to hematoxylin-eosin staining, immunohistochemical staining, flow cytometry analysis, and an EBV in situ hybridization analysis were performed for pathologic confirmation. The follow-up evaluation was established on the basis of the clinical follow-up, which included clinical examinations, ultrasonography, contrast-enhanced CT, ^18^F-FDG PET /CT scanning, or an additional biopsy every 1–3 months.

### CR definition

In this study, the patient outcome was defined as a CR when all lesions of MTX-LPD had disappeared after the withdrawal of MTX without cytotoxic treatment (i.e., chemotherapy and radiotherapy) during the follow-up. The patient outcome was defined as non-CR when additional chemotherapy was needed to treat the residual tumor after the withdrawal of MTX or the patient had residual tumor or a recurrence during the follow-up period. We defined the clinical outcome at the time of our review.

### PET/CT imaging

All patients underwent ^18^F-FDG PET/CT acquisitions using an integrated PET/CT scanner (Biograph 64 PET/CT scanner, Asahi-Siemens Medical Technologies, Tokyo). Before tracer injection, the patient fasted for at least 6 h. Following a blood glucose test to confirm blood glucose levels less than 150 mg/dL, PET images were acquired 60 min after an intravenous injection of ^18^F-FDG (4–5 MBq/kg). Emission scanning for 3 min per bed was carried out following the CT image acquisition for attenuation corrections.

The acquired datasets were corrected for attenuation, dead-time and scatter, and the images were reconstructed using a point spread function-based iterative algorithm (TrueX, Siemens) [18] with two iterations per 21 subsets with 512 × 512-pixel matrix, a matrix size of 168 × 168, a voxel size of 4.1 × 4.1 × 2.0 mm, and a Gaussian filter at 4.0- mm full-width at half-maximum. The transaxial and axial field of views were 58.5 cm and 21.6 cm, respectively.

### MDCT imaging

All patients were scanned with either a 64-slice or a 320-slice multi-detector row CT scanner. Six patients underwent a contrast-enhanced scan. These patients received an intravenous injection of iodine contrast agent (450–560 mgI/kg) at a rate of 2–3 mL/s using a power injector, and CT images were obtained approx. 60 s following the injection. The other nine patients underwent a non-contrast enhanced MDCT scan. The scan range of 12 patients was from the middle of the skull to the mid-thigh. The scan range of the other patients was the cervical region only, the abdominal region only, or from the cervical to chest region. Axial CT images were reconstructed using a 5-mm section thickness.

### Image analyses

Each patient’s disease stage was determined by the Ann Arbor classification system for malignant lymphoma [19]. Staging was confirmed by clinical follow-up or biopsy. We also divided the whole body into 16 nodal regions and nine extranodal anatomic regions for analysis, in accord with previous studies [20–22] (Table 2).

**Table 2:** Nodal and extranodal regions for region-based analysis

Nodal regions (*n* = 16)	Extranodal regions (*n* = 9)
Waldeyer ring	Upper aerodigestive tract
Right neck^a^	Skin/subcutaneous
Left neck^a^	Central nervous system and spinal canal
Right infraclavicular	Lung
Left infraclavicular	Myocardium
Right axillary and pectoral	Bone and bone marrow
Left axillary and pectoral	Liver
Mediastinal	Bowel
Hilar	Renal and adrenal
Spleen	
Paraaortic	
Mesenteric	
Right iliac	
Left iliac	
Right inguinal and femoral	
Left inguinal and femoral	

^a^Included cervical, supraclavicular, occipital, and preauricular regions

We compared each patient’s FDG-PET/CT findings with the results of the corresponding imaging examinations in terms of the presence of MTX-LPD disease. We classified the findings as follows. TP: true-positive (presence of MTX-LPD), TN: true-negative (absence of MTX-LPD), FP: false-positive (abnormal FDG uptake unrelated to MTX-LPD), or FN: false-negative (missed diagnosis of proved MTX-LPD) according to the reference standard. The reference standard included histopathologic findings (obtained by biopsy) or informative follow-up (clinical, laboratory, PET/CT, or other imaging findings such as endoscopy, MRI, and ultrasonography). It was impossible to obtain histopathologic proof of all of the suspected lesions in most of the clinical situations, especially in the patients with systemic disease spread. We therefore set the reference standard by using the method comparing PET/CT and MDCT findings in studies similar to ours [23, 24].

For example, in the case of a patient entering remission, lesions that were resolved on follow-up imaging were considered TP, and lesions that remained stable or progressed were considered FP. In the case of a patient experiencing disease progression, lesions that progressed were considered TP, and lesions that resolved or remained stable on follow-up imaging were considered FP. A region was considered FN if a lesion was not seen by one modality but was identified by another modality and met the criteria for a TP result as described above. A region was considered an FN if lesions were seen but were considered non-malignant on FDG-PET/CT or MDCT and disease developed at the same site of follow-up. Regions were considered TN when the site remained disease-free at the follow-up after MTX withdrawal.

The clinical images were independently evaluated in random order by two nuclear medicine physicians (S.W. and O.M.) for ^18^F-FDG PET/CT and two diagnostic radiologists (Y.K. and N.M.) for MDCT. The readers were blinded to all clinical information, the results of the other imaging modalities, such as endoscopy, MRI, and ultrasonography before MTX withdrawal, and the evaluation by the other reader.

Thirty-three lymph node regions and 18 extranodal anatomic regions were out of the field of view of the anatomical CT scanning. Therefore, a final total of 207 lymph node regions and 117 extranodal anatomic regions were analyzed. Areas with focally increased ^18^F-FDG uptake were considered to be sites of active disease for PET/CT. For the MDCT analysis, abnormal lymph nodes were defined as having a minimum dia. >1 cm and extranodal lesion as mass lesions considered as equivocal or positive for malignancy. All regions were evaluated as positive or negative. If there was a discordant finding between the two readers, consensus was obtained by discussion. Lesions considered positive, suggestive or equivocal for MTX-LPD were considered positive for the analysis. Lesions reported as unlikely or negative for MTX-LPD were considered negative for the analysis. The number of disease sites was evaluated by each physician.

In addition, one nuclear medicine physician (S.W.) assessed the ^18^F-FDG PET images using semi-quantitative methods. The SUVmax from the single pixel showing the highest FDG accumulation in the lesion was calculated to obtain the information of tumor activity, as [tissue radioactivity concentration (Bq/ml)] × [body weight (g)] / [injected radioactivity (Bq)]. Two volume-based parameters, the WBMTV and the WBTLG, were also measured.

The WBMTV was calculated from the ^18^F-FDG PET images according to the following procedure. First, the boundaries of voxels with an SUV intensity exceeding 2.5 were produced automatically. Second, the physiological uptake including that in the brain, oral cavity, pharynx, heart, stomach, liver, intestines, kidney, ureter, bladder, skeletal muscle, and any other tissues was carefully subtracted by the nuclear medicine physician. Third, false-positive lesions, such as inflammation of joints or other benign ^18^F-FDG-avid lesions based on MDCT or follow-up studies including physiological examination, MDCT, ultrasonography and PET/CT scan were subtracted. Finally, the total lesion glycolysis (TLG) value was calculated for every target lesion as TLG = MTV × SUVmean, and the WBTLG was calculated as the total of TLG in the entire body.

### Statistical analysis

Data are expressed as the median and range. Differences in the SUVmax, WBMTV and WBTLG between two groups were tested using Welch’s *t*-test for categorical variables. A Bowker test was used to compare the accuracy of ^18^F-FDG PET/CT scans and MDCTs in detecting malignant lesions. For each analysis, *p*-values <0.05 were considered significant. Statistical calculations were carried out using statistical software (JMP ver. 10, SAS, Cary, NC, USA).

## Results

### Diagnostic accuracy

MTX-LPD lesions were analyzed in 207 nodal regions and 117 extranodal regions and identified in 75 nodal anatomic regions (36.2 %) and 17 extranodal anatomic regions (14.5 %). ^18^F-FDG PET/CT was superior to MDCT at detecting both nodal and extranodal regions. ^18^F-FDG PET/CT scanning could detect 70/75 nodal (93.3 %) and 11/17 extranodal anatomic regions (64.7 %), whereas MDCT detected only 45/75 nodal (60.0 %) and 9/17 extranodal anatomic regions (52.9 %). Consequently, the sensitivity, specificity, and accuracy (Table 3.) for the detection of lesions were 83/92 (90.2 %), 226/232 (97.4 %), and 309/324 (95.4 %), respectively, for ^18^F-FDG PET/CT and 55/92 (59.8 %), 220/232 (94.8 %), and 275/324 (84.9 %), respectively, for MDCT (Table 4). The diagnostic accuracy of FDG-PET/CT was significantly higher than that of MDCT (*p* < 0.002). In the evaluation of interobserver agreement, discordant assessments were observed for five nodal regions and none of the extranodal regions in the FDG PET/CT reading, and for 30 nodal regions and five extranodal regions in the MDCT reading.

**Table 3:** Definitions of diagnostic test parameters

Diagnostic test parameter	Definition
Sensitivity	TP/(TP + FN)
Specificity	TN/(TN + FP)
Accuracy	(TP + TN)/(TP + FP + FN + TN)

*FN* false negative, *FP* false positive, *TN* true negative, *TP* true positive

**Table 4:** Comparison of ^18^F-FDG PET/CT and CT for the region-based detection of nodal and extranodal disease

	Region-based analysis					Diagnostic performance		
	TP	FP	TN	FN	Total	Sensitivity	Specificity	Accuracy
FDG-PET/CT	83	6	226	9	324	83/92 (90.2 %)	226/232 (97.4 %)	309/324 (95.4 %)
CT	55	12	220	37	324	55/2 (59.8 %)	220/232 (94.8 %)	275/324 (84.9 %)

*FN* false negative, *FP* false positive, *TN* true negative, *TP* true positive

**Fig. 1:**
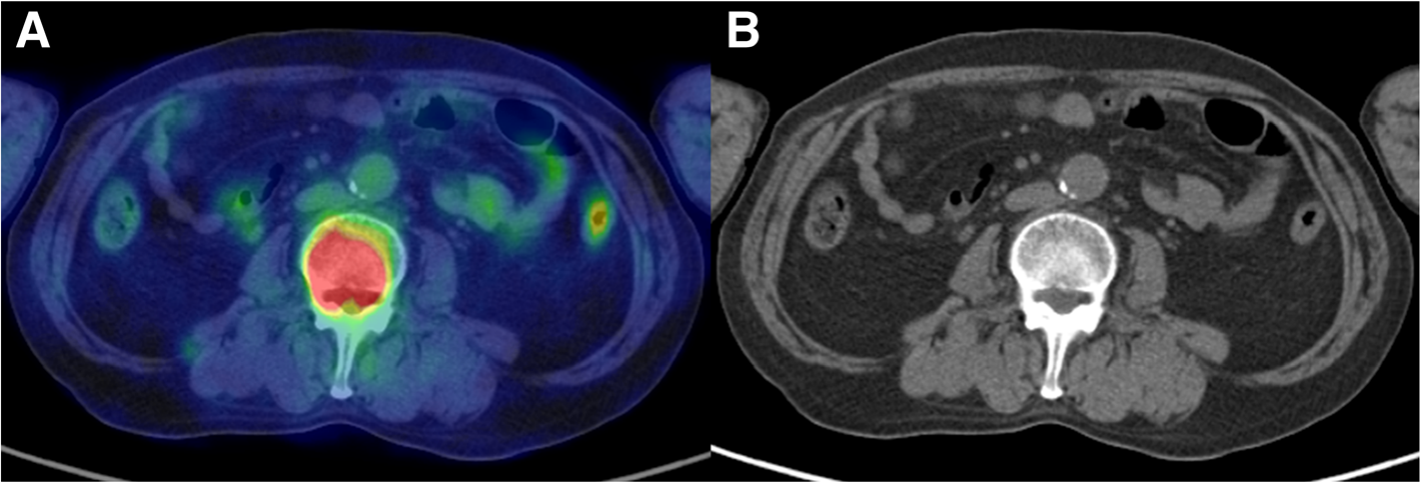
The PET/CT-positive and MDCT-negative case. Bone marrow involvement with obvious FDG uptake was detected by PET/CT (**a**). The MDCT scan showed no mass lesion (**b**)

There were five extranodal lesions that ^18^F-FDG PET/CT scanning could identify but MDCT could not; one in the bowel, one in bone (Fig. 1), one in the tonsil and two in the gingiva. The FN lesions were five nodal regions; both sides of the infraclavicular, the right axilla, and both sides of the inguinal and femoral regions. Four extranodal regions were FN; three in skin detected by only visual examination and one in lung. There were six FP lesions; lung, bone and four nodal lesions in the axilla, hilar, mesenteric, and iliac regions. One was a lung lesion that showed focal ^18^F-FDG uptake; this was pneumonitis. A second FP lesion was on bone that had focal ^18^F-FDG uptake, which was shown to be negative by bone marrow aspiration cytology. The other FPs identified by ^18^F-FDG PET/CT scanning were four nodal lesions that include the axilla, hilar, mesenteric and iliac regions with asymmetric ^18^F-FDG uptake.

### ^18^F-FDG PET parameters

The medians and ranges of the ^18^F-FDG PET parameters were as follows. SUVmax: 10.3 (range 0–47.1), WBMTV: 33.5 ml (range 0–362.6 ml), and WBTLG: 167.0 ml (range 0–2180.9 ml). The differences in the metabolic parameters between the CR patients and the non-CR patients are presented in Table 5. There was no significant association between ^18^F-FDG PET/CT parameters and the CR patients.

**Table 5:** Predictive value of each status

	CR (*n* = 9)	non-CR (*n* = 6)	*p*-value
Duration of MTX (month)	50 (7–172)	81 (40–234)	0.19
Dose of MTX (mg/week)	8.0 (4.0–16.0)	9.0 (6.0–16.0)	0.14
Number of sites	5 (1–99)	3 (1–41)	0.30
SUVmax	9.2 (2.8–47.1)	13.9 (0–24.9)	0.21
WBMTV (ml)	26.6 (0–362.6)	22.7 (0–250.1)	0.36
WBTLG (ml)	111.6 (0–2180.9)	152.1 (0–1052.1)	0.25
sIL-2R (U/ml)	758 (310–3157)	795 (259–5861)	0.16

*CR* complete response, *MTX* methotrexate, *sIL*-*2R* soluble interleukin-2 receptor, *SUVmax* maximum of standardized uptake value, *WBMTV* whole body metabolic tumor volume, *WBTLG* whole body total legion glycolysis

## Discussion

The results of this study demonstrated that the diagnostic accuracy of ^18^F-FDG PET/CT for identifying MTX-LPD lesions was significantly higher than that of MDCT scans, and we observed that none of the ^18^F-FDG PET parameters, i.e., SUVmax, WBMTV, and WBTLG could be used to distinguish responders from non-responders to the withdrawal of MTX.

^18^F-FDG PET/CT is widely accepted as the most sensitive imaging modality for staging, monitoring the response, and predicting the prognosis for various lymphomas [11–14, 25, 26]. The usefulness of ^18^F-FDG PET/CT scans for assessing MTX-LPD has been investigated [4–6]. Our present findings revealed that ^18^F-FDG PET/CT was more successful than MDCT in detecting nodal and extranodal malignant lesions, which is consistent with other types of lymphomas. The ^18^F-FDG PET/CT scans will correctly alter the initial disease stage for some patients and follow the distribution of lesions. These data support the important role of ^18^F-FDG PET/CT scans for the staging of MTX-LPD.

There were five extranodal lesions that only ^18^F-FDG PET/CT scanning could identify: one bowel, one bone (Fig. 1), one tonsil, and two gingival lesions. For staging patients with Hodgkin lymphoma or aggressive NHL, Schaefer et al. reported that PET/CT was more sensitive and specific than even contrast-enhanced CT, and they suggested that PET/CT without intravenous contrast media was sufficient [24]. In the present study, the protocols of the MDCT scans were not the same; some patients underwent non-contrast CT. However, even with a contrast agent, those were difficult to distinguish from normal structures by the anatomical CT scans, and the artifact from the prosthesis made the diagnostic reading of the oral cavity difficult.

Of the nine malignant lesions that ^18^F-FDG PET/CT scanning failed to detect, the five nodal regions were both sides of the infraclavicular, the right axilla and both sides of the inguinal and femoral regions, and the four extranodal regions were three skin sites detected by visual examination and one lung lesion which was difficult to detect by CT in the expiratory state. These lesions were too small for the detection of FDG uptake, or the uptake of these lesions was considered physiological uptake because of symmetrical distributions. The PET/CT scans also detected six FP lesions: lung, bone and four nodal lesions in the axilla, hilar, mesenteric, and iliac regions.

MTX withdrawal and observation for a short period should be considered in the initial management of patients who develop LPD while on MTX therapy, because more than half of the deaths were due to complications from chemotherapy and other causes (except disease progression) [15, 16]. In their review of the MTX-LPD literature, Ichikawa et al. noted that CR most commonly occurred within 4 weeks after MTX withdrawal, and they therefore proposed that observation for 1–2 months without any treatment but with withdrawal of immunosuppressant therapy is indicated if the patient’s general condition allows [16].

**Fig. 2:**
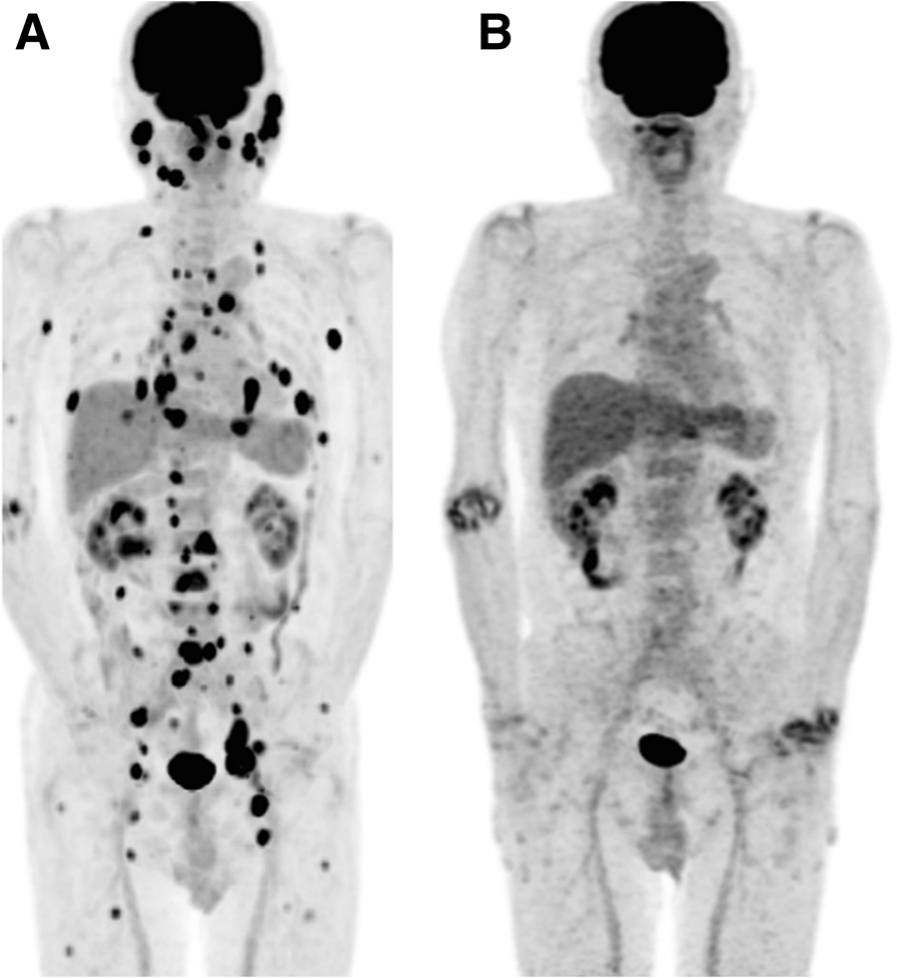
Representative case of complete response group. Maximum intensity projection images of ^18^F-FDG PET/CT at (**a**) initial examination, and (**b**) 4 months after from withdrawal of MTX were displayed. Multiple ^18^F-FDG -avid lesions was found in the whole body (SUVmax 9.2, WBMTV 44.3 ml, WBTLG 191.1 ml). The patient underwent ^18^F-FDG PET/CT again after cessation of MTX, which showed that multiple lesions on PET completely resolved. Regarding the metabolic activity of inflammation with Rheumatoid Arthritis, abnormal ^18^F-FDG uptake was appeared in large joints in a second PET/CT scans

**Fig. 3:**
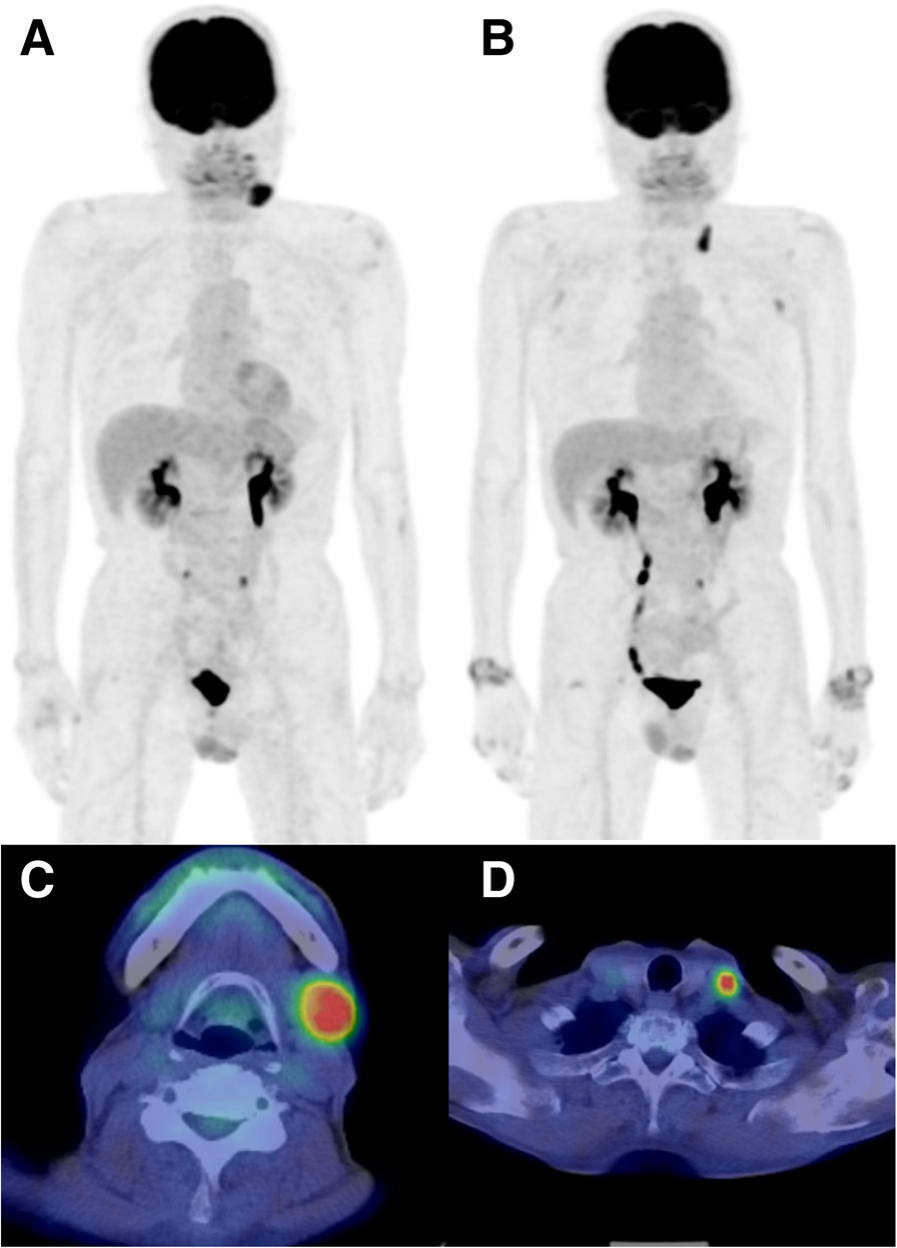
Representative case of non-complete response group. Maximum intensity projection images of ^18^F-FDG PET/CT at initial examination (**a**), and second examination at 6 months after MTX withdrawal (**b**) were shown. At the initial examination, an ^18^F-FDG-avid lesion was found at left neck (**c**; SUVmax 7.3, WBMTV 8.6 ml, WBTLG 42.7 ml). It was disappeared at the second examination after MTX withdrawal. However, at the second scan a recurrent lesion was found at left supraclavicular fossa (**d**) and radiotherapy was enforced. ^18^F-FDG uptake was confirmed at bilateral wrist joints, reflecting the reactivation of RA due to withdrawal of MTX

In the present analysis, nine patients achieved spontaneous remission of their LPD after MTX withdrawal (Fig. 2). Other studies also showed the potential usefulness of the MTV as a parameter for the prediction of clinical outcomes in several types of lymphoma [11, 12, 26, 27]. However, our present findings suggest that the metabolic activity could not predict the prognostic outcome when it comes to MTX-LPD, possibly because its behavior differs from that of other types of lymphoma (Fig. 3).

This study has some limitations. The retrospective nature of the study is a critical limitation in its generalization. The number of patients (*n* = 15) is small, because of the relatively low incidence of the disease. Another limitation is the use of clinical follow-up with additional imaging studies as the standard of reference, which may lead to a false classification of the lesions. Biopsies of every suggestive lesion are neither ethical nor recommended in routine clinical practice. Benign lesions, such as focal inflammation, that were resolved on follow-up imaging studies could have been falsely regarded as TPs. Conversely, malignant lesions with lower ^18^F-FDG-avidity could have been falsely regarded as negative. These misdiagnoses might have compromised the sensitivity of the ^18^F-FDG PET/CT scanning. Existing published studies share the same limitations [20, 28–31].

## Conclusion

^18^F-FDG PET/CT was a useful tool to detect MTX-LPD lesions. However, none of the ^18^F-FDG PET/CT parameters before MTX withdrawal could predict a CR after the withdrawal of MTX. MTX-LPD patients should be carefully monitored using follow-up ^18^F-FDG PET/CT regardless of the disease metabolic condition.

## Abbreviations

^18^F-FDG: ^18^F-Fluorodeoxyglucose
CT: Computed Tomography
EBV: Epstein-Barr Virus (EBV)
FN: False-negative
FP: False-positive
LPD: Lymphoproliferative Disorder
MDCT: Multi-detector Row CT
MTX: Methotrexate
NHL: Non-Hodgkin Lymphoma
PET: Positron Emission Tomography
RA: Rheumatoid Arthritis
SD: Standard Deviation
SUVmax: Maximum Standardized Uptake Value
TN: True-negative
TP: True-positive
WBMTV: Whole-body Metabolic Tumor Volume
WBTLG: Whole-body Total Lesion Glycolysis
WHO: World Health Organization
